# Application of metagenomics for diagnosis of broilers displaying neurological symptoms

**DOI:** 10.1186/s12917-023-03732-y

**Published:** 2023-10-05

**Authors:** Hyeon-Su Kim, Si-Hyeon Kim, Hye-Soon Song, Yong-Kuk Kwon, Choi-Kyu Park, Hye-Ryoung Kim

**Affiliations:** 1 Avian Disease Division, Animal and Plant Quarantine Agency, 177 Hyeoksin 8-ro, Gimcheon-si, 39660 Korea; 2 College of Veterinary Medicine & Animal Disease Intervention Center, Kyungpook National University, Daegu, 41566 Korea

**Keywords:** Chicken, Meningoencephalitis, Neurological symptom, Metagenomics, Histopathology

## Abstract

**Background:**

Thirty-two-day-old broiler chickens at a farm located in northwestern South Korea displayed adverse neurological symptoms including limping, lying down, and head shaking. Approximately 2.1% of chickens died or were culled due to severe symptoms. Five carcasses were submitted to the Avian Disease Division of the Animal and Plant Quarantine Agency (APQA) for disease diagnosis.

**Results:**

Broilers displayed severe pericarditis and perihepatitis associated with gross lesions. Broilers also displayed microscopic lesions in the cerebrum and in the granular layer of the cerebellum, which were associated with multifocal perivascular cuffing and purulent necrosis in the cerebrum, and severe meningitis with heterophil and lymphocyte infiltration. *Staphylococcus spp*. were identified in the liver and heart using bacteriological culture. PCR/RT-PCR assays revealed that broilers were negative for avian *Clostridium botulinum*, Newcastle disease virus, and avian encephalomyelitis virus. Bacterial and viral metagenomic analysis of brain sample further revealed the presence of *Pseudomonas spp.* and Marek’s disease virus, which are known etiological agents of chicken meningoencephalitis.

**Conclusions:**

This study reports a diagnostic analysis of gross and histopathological lesions from 32-day-old broilers displaying unique neurological symptoms that revealed the presence of the several neurological diseases including meningoencephalitis. The causative agents associated with meningoencephalitis of broilers that had not been identified by routine diagnostic methods could be diagnosed by metagenomics, which proves the usefulness of metagenomics as a diagnostic tool for unknown neurological diseases in broilers.

**Supplementary Information:**

The online version contains supplementary material available at 10.1186/s12917-023-03732-y.

## Background

Genomic identification and characterization of pathogenic microorganisms (bacteria, viruses, fungi, and parasites) isolated from various hosts and the environment play an important role in the diagnosis, monitoring, and control of infectious diseases [1, 2]. Though identification of causative agents by isolation and culture is recognized as the gold standard, these methods are labor-intensive, and require between 1 week to more than 6 weeks to provide a diagnosis [3]. Furthermore, many pathogens are difficult or impossible to culture using readily available technologies, hampering effective diagnostics [4].

PCR-based techniques are a highly sensitive, specific, and reproducible method to amplify target sequences to detect pathogens more quickly than classical culture methods [2]. However, these methods can target only a limited number of pathogens using specific primers or probes; this limits their application to detect newly emerging pathogens, which continually accumulate point mutations, genomic rearrangements, or recombination events. To overcome these limitations, metagenomics is becoming a widespread alternative method to conventional diagnostic techniques [5].

Early use of metagenomics for diagnostics includes the discovery of severe acute respiratory syndrome coronavirus (SARS-CoV), and genetic profiling of cancer mutations in the early 2000s [6–8]. Since 2005, Next Generation Sequencing (NGS) technologies have been increasingly applied to diagnostics in both humans and livestock, due to decreased sequencing cost and technological advancements. [9, 10]. In particular, the emergence of zoonotic diseases transmitted between humans and animals, such as SARS-CoV, Middle East Respiratory Syndrome coronavirus, or Coronavirus disease 2019, has led to widespread application of metagenomics in the field [11, 12].

Neurologic symptoms in broiler flocks between 4 and 5 weeks of age are uncommon. In chickens, neurological symptoms are usually caused by infectious agents such as highly pathogenic avian influenza virus (HPAIV), Newcastle disease virus (NDV), avian encephalitis virus (AEV), and *Clostridium botulinum* (*C. botulinum*). Infection of chickens with bacteria such as *Escherichia coli*, although uncommon, can cause septicemia or otitis media that may eventually present with neurological symptoms [13]. Furthermore, neurological symptoms can have non-microbial etiologies including use of veterinary pharmaceuticals such as anticoccidial drugs. This study presents a histopathological and metagenomic analysis of 32-day-old broiler chickens displaying neurological symptoms.

## Results

### Gross findings and analysis of histopathological lesions

**Fig. 1:**
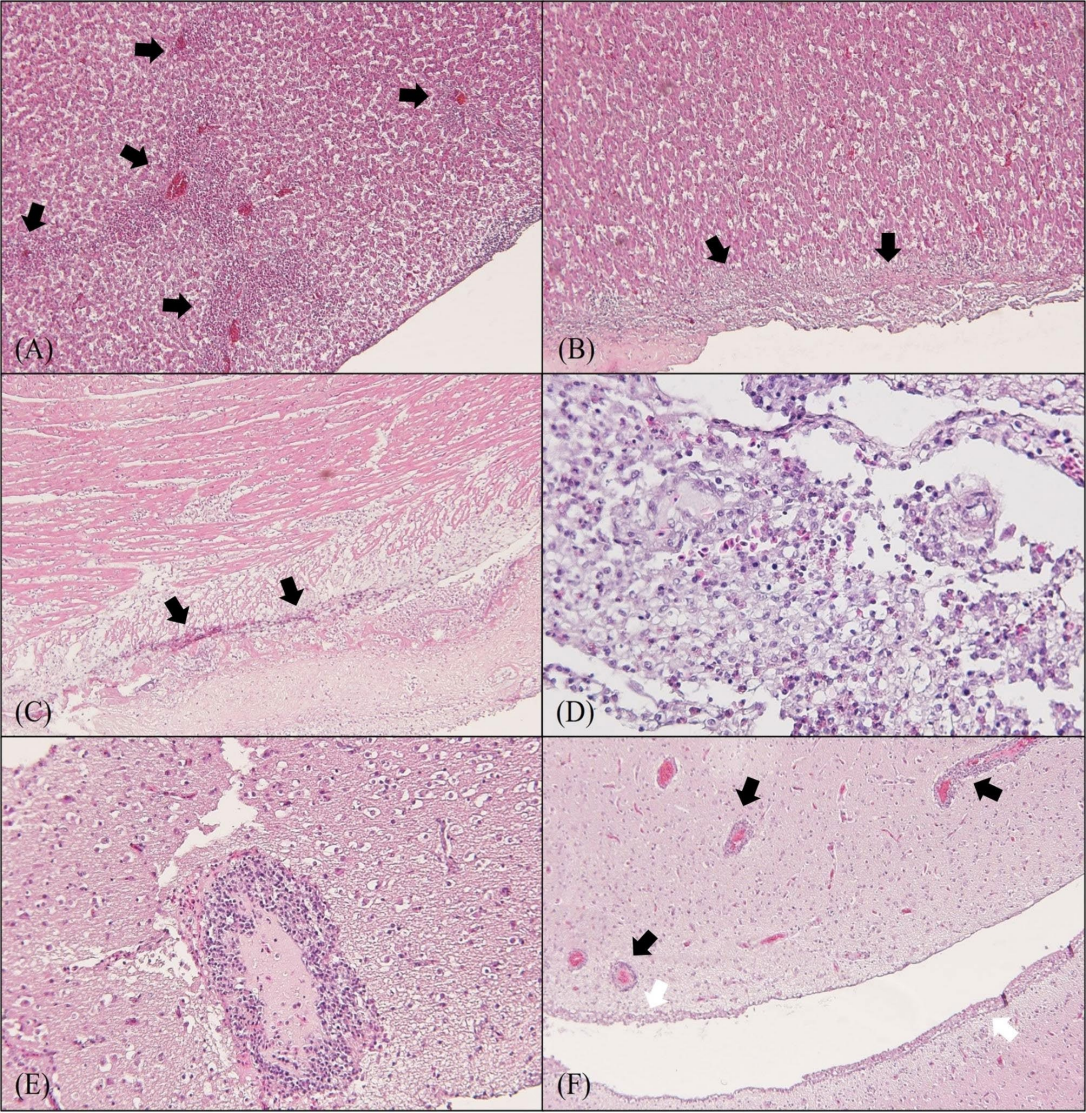
Histopathological lesions in the liver, heart, and brain of 32-day-old broiler chickens with neurological symptoms. **(A)** Acute hepatitis characterized by severely heterophilic and lymphocytic infiltration with sinusoid dilation (X100). **(B)** Serositis with fibrinous inflammation in the liver (X100). **(C)** Lymphocytic myocarditis and fibrinous pericarditis in the heart (X100). **(D)** Purulent meningitis in the brain (X400). **(E)** Heterophilic and lymphocytic encephalitis in the perivascular area of the brain (X200). **(F)** Perivascular cuffing (black arrow) and meningitis (white arrow) in the brain (X100)

Gross observation of symptomatic broilers revealed airsacculitis, pericarditis, and perihepatitis in three of five chickens. Histopathological analysis demonstrated presence of acute hepatitis with multifocal necrosis, moderate serositis, myocarditis and severe fibrinopurulent pericarditis in the liver and heart of chickens with gross lesions (Fig. 1A and B C). Furthermore, we observed suppurative meningitis associated with lymphocyte and heterophil infiltration, and meningeal proliferation in the meningeal pia mater of the cerebrum and cerebellum in the absence of gross lesions (Fig. 1D). We also observed encephalitis with lymphocytosis in the cerebral cortex (Fig. 1E). Interestingly, we identified moderate multifocal mononuclear cellular infiltration, consisting mainly of lymphocytes and monocytes, in the perivascular areas of cerebral cortex, which are usually observed in the case of viral encephalitis (Fig. 1F).

### Presumptive diagnosis

Following a traditional diagnostics protocol [14, 15], we isolated *Staphylococcus chromogense* and *Staphylococcus cohnii* from liver and heart samples, respectively, which also contained histopathological lesions. However, these pathogens are not suggested as causative agents of neurological symptoms. All samples were negative for the common etiological agents of neurological symptoms, HPAIV, NDV, AEV, and *C. botulinum*, by PCR and RT-PCR (Additional file 1: Table S1). Histopathological analysis of the brain sample from this flock identified signatures of bacterial meningoencephalitis and viral encephalitis, but the causative agent was unclear. We therefore applied metagenomics to identify the etiological agent of meningoencephalitis.

### Metagenomic data analysis

**Fig. 2:**
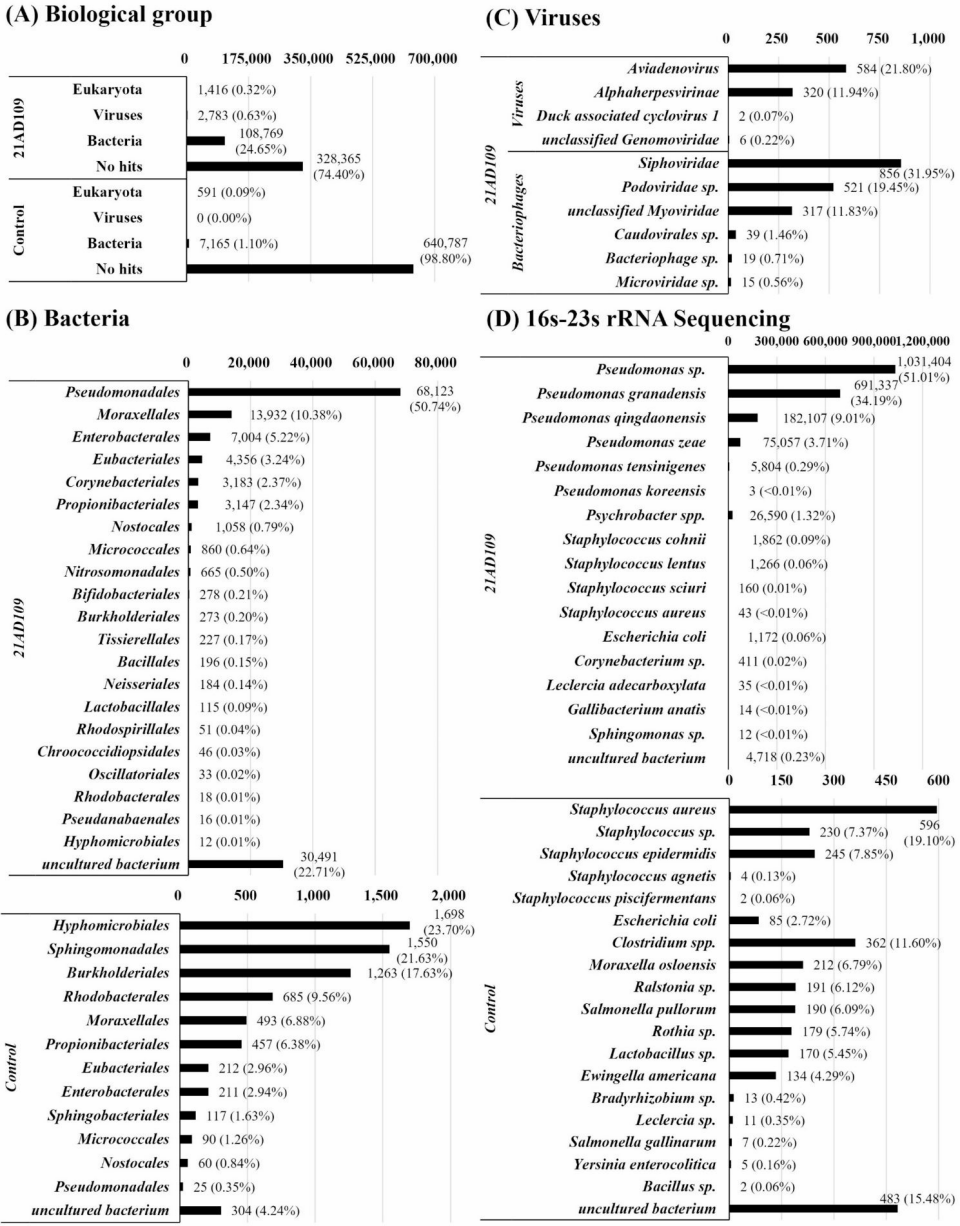
Results of viral metagenomic and 16-23 s rRNA sequencing. **(A)** Metagenomic data from the Miniseq system revealed four biological groups. **(B)** *Pseudomonadales* was the most frequent taxon in the bacterial group. **(C)** The viral group consisted of avian associated viruses and bacteriophages. The control sample included no viral reads. **(D)** Results of 16-23 s rRNA sequencing demonstrated the presence of *Pseudomonas spp.* including *Pseudomonas sp.*, *Pseudomonas granadensis*, and *Pseudomonas qingdaonensis*

A total of 16,577,781 raw reads were obtained from viral metagenomic data, with 91.3% having a Q score ≥ 30. After trimming and filtering adapters, primers, and low-quality reads, 16,266,961 reads remained, which were used to generate 217,921 contigs. Contigs were subsequently analyzed using BLAST probing the nr_euk database and classified into four biological groups: bacteria (108,769 reads, 24.65%), viruses (2,783 reads, 0.63%), eukaryotes (1,416 reads, 0.32%, except for *Gallus gallus*), and no hits (328,365 reads, 74.40%) (Fig. 2A). Among the 108,769 bacterial reads, the highest proportion encoded sequences from *Pseudomonadales* (68,123 reads, 50.74% of bacteria) (Fig. 2B). The distribution of bacteriophages and other viruses in the 2,783 viral reads was 65.96% and 34.04%, respectively. Identified viral sequences were assigned to known viral families, including *Aviadenovirus* (584 reads, 21.80%), *Alphaherpesvirinae* (320 reads, 11.94%), *Duck associated cyclovirus* I (2 reads, 0.07%), and *unclassified Genomoviridae* (6 reads, 0.22%). Bacteriophage comprised 38.7% of viral sequences and were assigned as *Siphoviridae*, *Podoviridae sp., unclassified Myoviridae, Caudovirales sp., Bacteriophage sp.* and *Microviridae sp* (Fig. 2C).

The control sample generated 12,044,235 reads and 94.1% had a Q score ≥ 30. After trimming and QC, 11,872,300 reads were assembled into 20,881 contigs. We removed the host genome from the final consensus, and the remaining 9,658 contigs and 441,333 mapped reads were classified into three biological groups: no hits (640,787 reads, 98.80%), bacteria (7,165 reads, 1.10%), and eukaryote (591 reads, 0.09%, except for *Gallus gallus*). No viral sequences were detected in the control sample (Fig. 2A).

We performed 16-23 s rRNA sequencing to identify bacterial species present in the clinical sample. Among the 2,023,995 reads of length 2–3 kb, *Pseudomonas spp.* were most frequent compared with other bacteria (1,985,712 reads, 98.2%). These reads mapped to *Pseudomonas sp.* (1,031,404 reads, 51.01%), *Pseudomonas granadensis* (691,337 reads, 34.19%), *Pseudomonas qingdaonensis* (182,107 reads, 9.01%), *Pseudomonas zeae* (75,057 reads, 3.71%), *Pseudomonas tensinigenes* (5,804 reads, 0.29%), and *Pseudomonas koreensis* (3 reads, < 0.01%) (Fig. 2D).

From 16 to 23 s rRNA sequencing of the control sample, we selected 3,121 reads for analysis. *Staphylococcus spp.* were most frequent compared with other bacteria (1,077 reads, 34.5%), followed by *Clostridium spp.* (362 reads, 11.60%), *Moraxella osloensis* (212 reads, 6.79%), and *Ralstonia sp.* (191 reads, 6.12%) (Fig. 2D).

### Phylogenetic analysis

Reads for the common chicken pathogens Marek’s disease virus (MDV) and Fowl Adenovirus (FadV) were detected using viral metagenomic analysis. As the yield of reads was insufficient to confirm their genetic characteristics, we performed Sanger sequencing of target genes from both viruses.

**Fig. 3:**
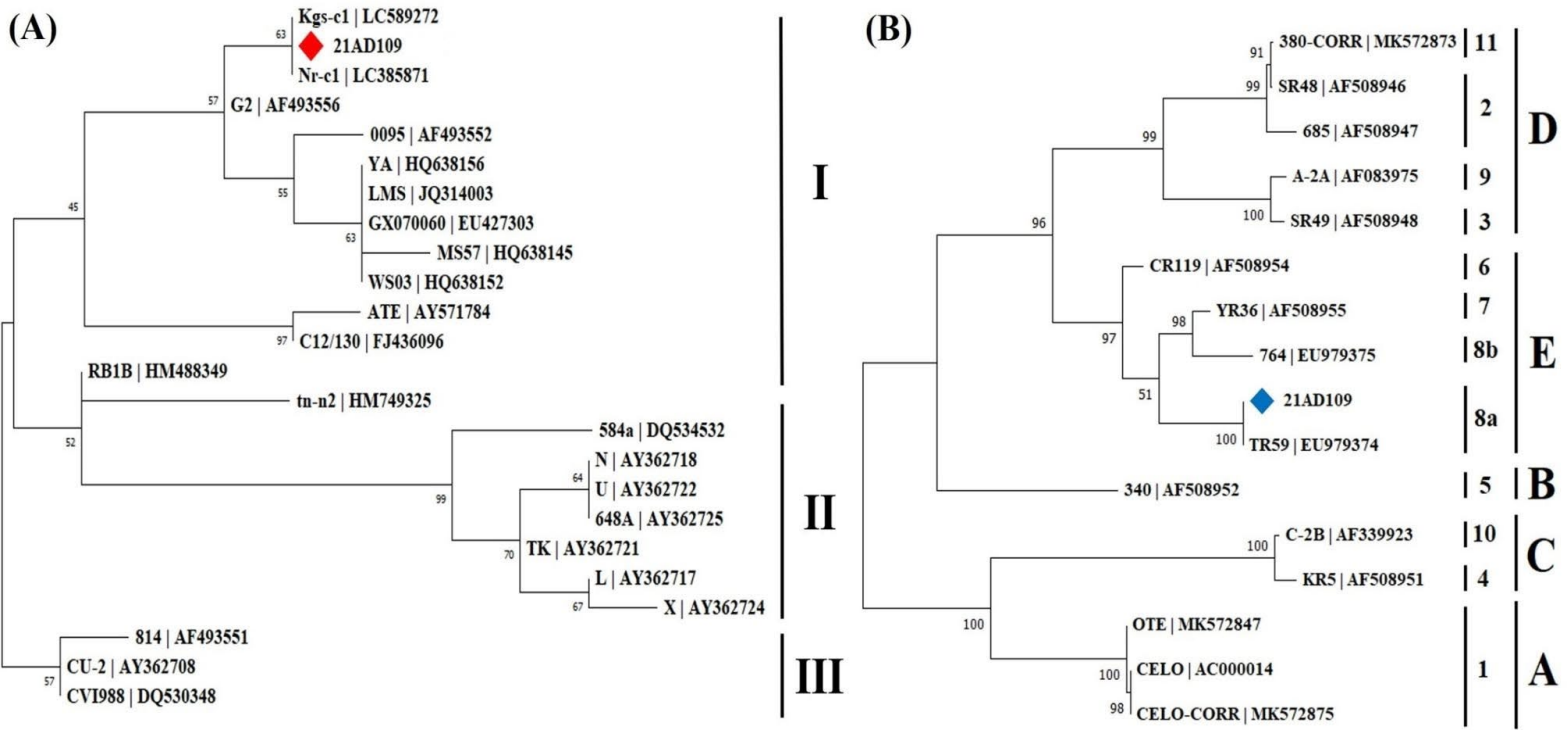
Phylogenetic tree of the two avian viruses identified in this study. **(A)** A phylogenetic tree constructed through neighbor joining methods with 1,000 bootstrap replicates using the *meq* gene of MDV (◆: tested case). **(B)** A phylogenetic tree generated through maximum likelihood methods with 1,000 bootstrap replicates using the Loop-1(L1) region of the *hexon* gene of FadV (◆: tested case)

In order to discriminate whether the obtained MDV sequences are from field or vaccine strains (CVI988, AF493551), partial sequences (short *meq* : 858 nt, long *meq* : 1035 nt) of the *meq* gene were aligned with 23 reference strains, and a phylogenetic tree was generated consisting of three groups. The *meq* gene of Group I and II was 177 bp shorter than that of group III, which consisted of vaccinal strains. Group I contained two branches, divided into the European virulent (ATE, C12/130) and Chinese virulent strains (LMS, YA, WS03, GX070060, G2). MDV strains identified in the 21AD109 sample were closely related to Kgs-c1 and Nr-c1 strains with 100% nucleotide identity in group I, known as the Japanese strain (Fig. 3A).

We also aligned a partial sequence (494 nt) of the *hexon* gene from FadV with 15 reference strains. Phylogenetic analysis revealed that the FadV in the 21AD109 sample was closely related to the TR59 strain with 100% sequence identity to serotype 8a(E) (Fig. 3B).

## Discussion

Suppurative meningitis in the cerebrum and cerebellum is caused by bacterial infection of the central nervous system (CNS), accessed through the blood stream. We identified *P. granadensis* from the brain sample of broilers with suppurative meningitis by metagenomic sequencing of 16-23 s rRNA, suggesting that it may be the causative agent of suppurative meningitis of the broilers. *P. granadensis* was previously isolated from soil in Spain in 2015 and belongs to the *P. fluorescens* lineage [16]. *Pseudomonas* are a ubiquitous genus of bacteria, commonly found in a wide variety of environments, and are also opportunistic pathogens that cause infections in immunocompromised individuals [17, 18]. Therefore, it is suggested that *P. granadensis* caused an opportunistic infection due to immunosuppression by MDV.

To infect the CNS, bacteria should first pass through the blood-brain barrier (BBB). Pathogens can cross the BBB transcellularly, paracellularly, and/or by Trojan-horse mechanisms [19]. Studies on these infection modalities have been conducted using several strains in both in vivo and in vitro models [20–23]. Pathogens can also cause BBB dysfunction by interfering with the expression of host factors such as chemokines or cytokines that affect cell adhesion, or by inducing cytotoxicity and apoptosis. These observations imply that all bacteria capable of crossing the BBB by these mechanisms have the potential to cause meningitis.

Our histopathological analysis revealed cuffing in the perivascular area of the brain, which is a typical lesion caused by viruses such as viral encephalitis [24]. Viral metagenomic analysis identified MDV and FadV, and phylogenetic analysis confirmed the virulence profile and serotype of strains in clinical samples.

Marek’s disease (MD) is a highly oncogenic, lymphoproliferative, immunosuppression, and neuropathic disease [25]. Transient paralysis (TP) is acutely induced by MDV within 24–48 h and can lead to either mortality or recovery from early paralytic symptoms. Conversely, in our unique case, mortality occurred continuously for six days in a broiler flock. Histopathological signs of infection in the brain in conjunction with neurological symptoms such as paralysis after MDV infection have only been observed experimentally using in vivo models, and not in the field [26, 27]. According to the author’s knowledge, our study may be the first recorded case of meningoencephalitis in broiler using metagenomics in the field.

FadV is separated into five genotypes (A-E) and 12 serotypes (1–12), some of which can cause inclusion body hepatitis (IBH), hepatitis hydropericardium syndrome (HHS), and gizzard erosion (GE) [28]. Since we observed no lesions related to FadV in this study, this virus is an unlikely causative agent of neurological symptoms. We may have detected FadV because it is commonly found in poultry farms.

Since the advent of NGS technologies, sequencing has become faster, cheaper, and more accurate. The development of these technologies has enabled comprehensive analysis using metagenomics and can now be applied to clinical samples. Since conventional methods such as PCR or RT-PCR can diagnose only a limited number of known pathogens, metagenomics will be useful for detecting unknown and emerging pathogens that are not routinely tested in diagnostic laboratories. As diseases such as encephalitis that originate in the CNS have a wide range of causative agents, the application of metagenomics rather than standard methods will be more effective for accurate diagnosis.

## Conclusions

We performed a standard diagnosis based on gross and histopathological lesions in addition to a metagenomics to diagnose a unique case of mortality associated with neurological symptoms in 32-day-old chickens. We isolated *S. chromogense* and *S. cohnii*, the causes of pericarditis and perihepatitis, from the liver and heart using traditional culture methods, and identified causative agents of meningoencephalitis using metagenomics.

**Fig. 4:**
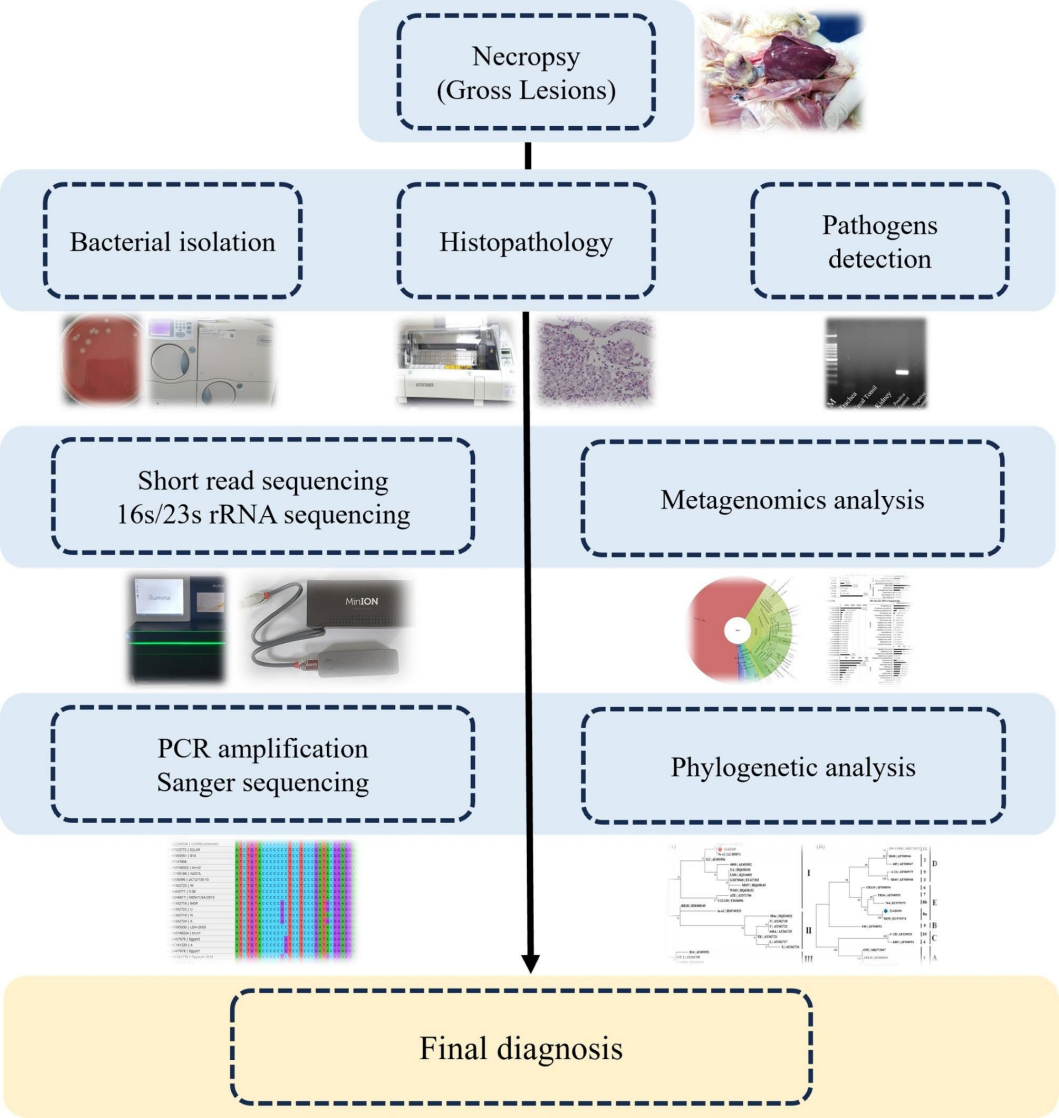
Schematic of diagnostic process using traditional and metagenomics methods

Our study may be the first field report demonstrating MDV and bacterial co-infection associated with clinical symptoms and histopathological lesions based on the author’s knowledge. The combined use of metagenomics and traditional culture methods represent a paradigm shift in diagnostics. Our flowchart of diagnostic process may help people understand better to combine with metagenomics and traditional methods (Fig. 4).

## Methods

### Samples

Five carcasses from a farm located in northwestern South Korea were submitted to the Avian Disease Division of the Animal and Plant Quarantine Agency (APQA) for diagnosis in 2021. Neurological symptoms including limping, lying down and head shaking were observed in a flock of 32-day-old broiler chickens, resulting in 2.1% of this flock either dying or culled across a 6-day periods. As a results, gross lesions were characterized, and we performed necropsy, bacteriological culture, and virus isolation using specific-pathogen-free chicken embryonated eggs, following APQA diagnostic protocols [14]. The trachea, cecal tonsils, kidneys, livers, and brains were collected from 5 broilers, and pooled by each organ. After necropsy, samples were named 21AD109. Specific-pathogen-free chicken brains were used as a control. The collected organs were fixed for 24 h in 10% neutral-buffered formalin for histopathological analysis. The remaining organs were homogenized in 10% phosphate-buffered saline and stored at -70℃ until further processing.

### Bacterial examination

The surface of livers and hearts were seared with a hot spatula, and samples were obtained using a sterile swab. Swap samples were subsequently cultured on blood agar and MacConkey agar plates. After incubation overnight at 37℃, single colony was selected, and species identity was determined using the Vitek 2 system (bioMérieux, France) according to the manufacturer’s instructions.

### Genome samples preparation and PCR amplification

Total bacterial and viral genomic nucleic acid was isolated from homogenized samples using a Maelstrom 4000 DNA/RNA auto-extraction machine (TAN bead, Taiwan). PCR and RT-PCR assays were conducted using the specific primer pairs for *C. botulinum*, HPAIV, AEV and NDV as reported in previous studies [29–31]. PCR products were analyzed under UV light after separation by 1.5% agarose gel electrophoresis.

### Histopathological examination

Fixed tissue samples were trimmed to an appropriate size for the fixative container and washed in running water for 30 min to remove excess formalin. Trimmed tissues were dehydrated using a STP120 Spin Tissue Processor (Thermo-Fisher Scientific, USA) and embedded in molten paraffin using an embedding cassette (HYUNIL Lab-Mate, Korea). Section 5 μm thick were rehydrated with xylene before staining with hematoxylin and eosin (H&E) in a ST5010 Autostainer XL (Leica Biosystems, Germany). Finally, stained tissues were mounted on glass slides and were analyzed for the presence of histopathological lesions using a microscope.

### Bacterial and viral metagenomic sequencing

Homogenized brain sample was centrifuged at 800×g for 5 min and 13,000×g for 10 min, respectively. Supernatants for viral metagenomic sequencing were passed through a 0.45 μm polyethersulfone membrane filter (TPP, Switzerland) to remove residual bacteria and debris. Virus in the residual supernatant was precipitated with 8% polyethylene glycol 6000 and 0.5 M NaCl, as previously described [32, 33]. Genomic DNA/RNA was extracted using a Maelstrom 4000 DNA/RNA auto-extraction machine (TANbead, Taiwan). The extracted DNA/RNA sample was divided into two parts and treated with DNase I and RNase A for viral DNA/RNA enrichment, respectively, according to the manufacturer’s protocols (QIAGEN, Germany). First strand cDNA synthesis was performed using PrimeScript™ 1st strand cDNA Synthesis Kit (Takara, Japan) according to the manufacturer’s instructions. Viral DNA and cDNA were amplified using a random primer (5′-GCC GGA GCT CTG CAG AAT TCN NNN NN-3′) with a LongAmp® Taq 2X Master Mix (NEB, USA) [34]. Illumina library preparation was performed using a Nextera XT DNA Library Preparation kit (Illumina, USA) according to the manufacturer’s recommendations. Libraries were quantified using a Qubit fluorometer (Invitrogen, USA) and 4150 TapeStation system (Agilent Technologies, USA). Libraries were denatured and sequenced on an Illumina MiniSeq with a MiniSeq Reagent kit (Illumina, USA).

Homogenized brain sample for 16-23 s rRNA sequencing were amplified with previously modified primers 27 F (5′-AGA GTT TGA TCC TGG CTC AG-3′) and 2490R (5′-GAC ATC GAG GTG CCA AAC-3′) using the Maxime PCR PreMix Kit (Intron, Korea) with the following conditions: 5 min at 94 °C, followed by 30 cycles of 30 s at 94 °C, 30 s at 57 °C, and 1 min at 72 °C, and a final extension step at 72 °C for 10 min [35]. The amplified products were purified using a QIAquick PCR purification Kit (QIAGEN, Germany) and an Oxford nanopore library was prepared using SQK-LSK109, following the manufacturer’s protocol (Genomic DNA by Ligation, ONT). Sequencing was performed with a flowcell (R9.4) on the MinION MkIB for 12 h.

### Bioinformatics analysis

For analysis of viral metagenomics, the extracted paired-end reads were trimmed using Cutadapt (V2.8) and Trimmomatic (V0.39) [36, 37]. The resulting reads were assembled using the metaSPAdes mode of the SPAdes software (V3.15.4) and mapped to a *Gallus gallus* reference genome (GCF_016699485) using Bowtie2 (V2.4.5) to remove chicken genomic sequence [38, 39]. Reads were classified using the Kaiju taxonomic classification program (V1.8.2) with the NCBI nr database including eukaryotes, and graphically represented using a Krona Chart [40, 41].

The long-read 16-23 s rRNA data was trimmed of reads except those of length 2–3 kb using Fastp (V0.20.1) [42]. The host genome and chimeras of selected reads were automatically removed using Bowtie2 (V2.4.5) and Vsearch (V2.21.1), respectively [41, 43]. Finally, the remaining 16-23 s rRNA reads were classified with Mothur (V1.48.0) [44].

### Phylogenetic analysis

To validate the viral metagenomic data, extracted DNA/RNA was subject to PCR amplification targeting the *meq* gene of MDV *and hexon* gene of FadV, in line with previous studies [45, 46]. PCR amplification of MDV was performed in a 20 µL total reaction volume with a 2×Black PCR premix (Ventech Science, South Korea), 2.0 µL of primers, and 2.0 µL of template DNA. Conditions for amplification of MDV were as follows: initial denaturation at 94 °C for 5 min, 38 cycles of denaturation at 94 °C for 45 s, annealing at 55 °C for 45 s, and extension at 72 °C for 45 s, and a final extension at 72 °C for 5 min. The amplicon was purified and sequenced by Macrogen (Daejeon, South Korea). Sequences were aligned using Clustal W and phylogenetic trees were constructed using a maximum likelihood method with 1,000 bootstrap replicates in Mega X (V.10.2.6) [47].

### Electronic supplementary material

Below is the link to the electronic supplementary material.

Supplementary Material 1

## Data Availability

The datasets generated in this study are available in the NCBI Sequence Read Archive (SRA) under BioProject: PRJNA954528 https://www.ncbi.nlm.nih.gov/bioproject/PRJNA954528.

## Abbreviations

SARS-CoV: Severe Acute Respiratory Syndrome Coronavirus
HPAIV: Highly Pathogenic Avian Influenza Virus
NDV: Newcastle Disease Virus
AEV: Avian Encephalitis Virus
MDV: Marek’s Disease Virus
FadV: Fowl Adenovirus
CNS: Central Nervous System
BBB: Blood-Brain Barrier
MD: Marek’s Disease
TP: Transient Paralysis
IBH: Inclusion Body Hepatitis
HHS: Hepatitis Hydropericardium Syndrome
GE: Gizzard Erosion

## References

[1] Peker N, Garcia-Croes S, Dijkhuizen B, Wiersma HH, van Zanten E, Wisselink G, Friedrich AW, Kooistra-Smid M, Sinha B, Rossen JWA, Couto N (2019). A comparison of three different Bioinformatics analyses of the 16S-23S rRNA Encoding Region for bacterial identification. Front Microbiol.

[2] Qiu Y, Wang S, Huang B, Zhong H, Pan Z, Zhuang Q, Peng C, Hou G, Wang K (2019). Viral infection detection using metagenomics technology in six poultry farms of eastern China. PLoS ONE.

[3] Salipante SJ, Sengupta DJ, Rosenthal C, Costa G, Spangler J, Sims EH, Jacobs MA, Miller SI, Hoogestraat DR, Cookson BT, McCoy C, Matsen FA, Shendure J, Lee CC, Harkins TT, Hoffman NG (2013). Rapid 16S rRNA next-generation sequencing of polymicrobial clinical samples for diagnosis of complex bacterial infections. PLoS ONE.

[4] Didelot X, Bowden R, Wilson DJ, Peto TEA, Crook DW (2012). Transforming clinical microbiology with bacterial genome sequencing. Nat Rev Genet.

[5] Motro Y, Moran-Gilad J (2017). Next-generation sequencing applications in clinical bacteriology. Biomol Detect Quantif.

[6] Rota PA, Oberste MS, Monroe SS, Nix WA, Campagnoli R, Icenogle JP, Peñaranda S, Bankamp B, Maher K, Chen MH, Tong S, Tamin A, Lowe L, Frace M, DeRisi JL, Chen Q, Wang D, Erdman DD, Peret TC, Burns C, Ksiazek TG, Rollin PE, Sanchez A, Liffick S, Holloway B, Limor J, McCaustland K, Olsen-Rasmussen M, Fouchier R, Günther S, Osterhaus AD, Drosten C, Pallansch MA, Anderson LJ, Bellini WJ (2003). Characterization of a novel coronavirus associated with severe acute respiratory syndrome. Science.

[7] Sotiriou C, Pusztai L (2009). Gene-expression signatures in breast cancer. N Engl J Med.

[8] Chiu CY, Miller SA (2019). Clinical metagenomics. Nat Rev Genet.

[9] Brown JR, Bharucha T, Breuer J (2018). Encephalitis diagnosis using metagenomics: application of next generation sequencing for undiagnosed cases. J Infect.

[10] Kwok KTT, Nieuwenhuijse DF, Phan MVT, Koopmans MPG (2020). Virus Metagenomics in Farm Animals: a systematic review. Viruses.

[11] Ko KKK, Chng KR, Nagarajan N (2022). Metagenomics-enabled microbial surveillance. Nat Microbiol.

[12] Sawyer A, Free T, Martin J (2021). Metagenomics: preventing future pandemics. Biotechniques.

[13] Swayne DE, Boulianne M, Logue CM, McDougald LR. Venugopal Nair, David L. Suarez. Diseases of Poultry, 14th Edition. Wiley & Sons; 2019 Nov. p. 770–830.

[14] Animal and Plant Quarantine Agency (APQA). Standard guidelines for animal diagnosis. APQA Article. 2017: https://www.qia.go.kr/viewwebQiaCom.do?id=43875&type=2_102. Accessed 24 Jan 2018.

[15] World Organisation for Animal Health (WOAH). Manual of Diagnostic Tests and Vaccines for Terrestrial Animals 2022: https://www.woah.org/en/what-we-do/standards/codes-and-manuals/terrestrial-manual-online-access. Accessed 12 Jan 2022.

[16] Pascual J, García-López M, Bills GF, Genilloud O (2015). Pseudomonas granadensis sp. nov., a new bacterial species isolated from the Tejeda, Almijara and Alhama Natural Park, Granada, Spain. Int J Syst Evol Microbiol.

[17] Sitaraman R (2015). Pseudomonas spp. as models for plant-microbe interactions. Front Plant Sci.

[18] Abd El-Ghany WA (2021). *Pseudomonas aeruginosa* infection of avian origin: zoonosis and one health implications. Vet World.

[19] Kim KS (2008). Mechanisms of microbial traversal of the blood-brain barrier. Nat Rev Microbiol.

[20] van Sorge NM, Doran KS (2012). Defense at the border: the blood-brain barrier versus bacterial foreigners. Future Microbiol.

[21] Greiffenberg L, Goebel W, Kim KS, Daniels J, Kuhn M (2000). Interaction of Listeria monocytogenes with human brain microvascular endothelial cells: an electron microscopic study. Infect Immun.

[22] Wickham ME, Brown NF, Provias J, Finlay BB, Coombes BK (2007). Oral infection of mice with Salmonella enterica serovar typhimurium causes meningitis and infection of the brain. BMC Infect Dis.

[23] Baldissera MD, Souza CF, Santos RCV, Baldisserotto B (2018). Blood-brain barrier breakdown and myeloperoxidase activity in silver catfish experimentally infected with Pseudomonas aeruginosa. J Fish Dis.

[24] Oevermann A, Botteron C, Seuberlich T, Nicolier A, Friess M, Doherr MG, Heim D, Hilbe M, Zimmer K, Zurbriggen A, Vandevelde M (2008). Neuropathological survey of fallen stock: active surveillance reveals high prevalence of encephalitic listeriosis in small ruminants. Vet Microbiol.

[25] Swayne DE, Boulianne M, Logue CM, McDougald LR. Venugopal Nair, David L. Suarez. Diseases of Poultry, 14th Edition. Wiley & Sons; 2019 Nov. p. 550–587.

[26] Cho KO, Endoh D, Qian JF, Ochiai K, Onuma M, Itakura C (1998). Central nervous system lesions induced experimentally by a very virulent strain of Marek’s disease virus in Marek’s disease-resistant chickens. Avian Pathol.

[27] Gimeno IM, Witter RL, Reed WM. Four distinct neurologic syndromes in Marek’s disease: effect of viral strain and pathotype. Avian Dis 1999 Oct-Dec;43(4):721–37. PMID: 10611988. 10611988

[28] Swayne DE, Boulianne M, Logue CM, McDougald LR. Venugopal Nair, David L. Suarez. Diseases of Poultry, 14th Edition. Wiley & Sons; 2019 Nov. p. 321–339.

[29] Jang I, Lee JI, Kwon YK, Kang MS, Kim HR, Park JY, Lee SH, Lee HS, Bae YC (2015). Single-tube nested PCR assay for the detection of avian botulism in cecal contents of chickens. Anaerobe.

[30] Fouchier H, Bestebroer RA, Herfst TM, Van Der Kemp S, Rimmelzwaan L, Osterhaus GF (2000). Detection of influenza a viruses from different species by PCR amplification of conserved sequences in the matrix gene. J Clin Microbiol.

[31] Marvil P, Knowles NJ, Mockett AP, Britton P, Brown TD, Cavanagh D. Avian encephalomyelitis virus is a picornavirus and is most closely related to hepatitis A virus. J Gen Virol. 1999;80 (Pt 3):653–662. 10.1099/0022-1317-80-3-653. PMID: 10092005. 10.1099/0022-1317-80-3-653 10092005

[32] Yamamoto KR, Alberts BM, Benzinger R, Lawhorne L, Treiber G. Rapid bacteriophage sedimentation in the presence of polyethylene glycol and its application to large-scale virus purification. Virology. 1970;40(3):734 – 44. 10.1016/0042-6822(70)90218-7. PMID: 4908735. 10.1016/0042-6822(70)90218-7 4908735

[33] Veronese FM, Mero A. The impact of PEGylation on biological therapies. BioDrugs. 2008;22(5):315 – 29. 10.2165/00063030-200822050-00004. PMID: 18778113. 10.2165/00063030-200822050-00004 18778113

[34] Froussard P (1992). A random-PCR method (rPCR) to construct whole cDNA library from low amounts of RNA. Nucleic Acids Res.

[35] Sabat AJ, van Zanten E, Akkerboom V, Wisselink G, van Slochteren K, de Boer RF, Hendrix R, Friedrich AW, Rossen JWA, Kooistra-Smid AMDM (2017). Targeted next-generation sequencing of the 16S-23S rRNA region for culture-independent bacterial identification - increased discrimination of closely related species. Sci Rep.

[36] Martin M (2011).

[37] Bolger AM, Lohse M, Usadel B (2014). Trimmomatic: a flexible trimmer for Illumina sequence data. Bioinformatics.

[38] Nurk S, Meleshko D, Korobeynikov A, Pevzner PA (2017). metaSPAdes: a new versatile metagenomic assembler. Genome Res.

[39] Langmead B, Salzberg SL (2012). Fast gapped-read alignment with Bowtie 2. Nat Methods.

[40] Menzel P, Ng KL, Krogh A (2016). Fast and sensitive taxonomic classification for metagenomics with Kaiju. Nat Commun.

[41] Ondov BD, Bergman NH, Phillippy AM (2011). Interactive metagenomic visualization in a web browser. BMC Bioinformatics.

[42] Chen S, Zhou Y, Chen Y, Gu J (2018). Fastp: an ultra-fast all-in-one FASTQ preprocessor. Bioinformatics.

[43] Rognes T, Flouri T, Nichols B, Quince C, Mahé F (2016). VSEARCH: a versatile open source tool for metagenomics. PeerJ.

[44] Schloss PD, Westcott SL, Ryabin T, Hall JR, Hartmann M, Hollister EB, Lesniewski RA, Oakley BB, Parks DH, Robinson CJ, Sahl JW, Stres B, Thallinger GG, Van Horn DJ, Weber CF (2009). Introducing mothur: open-source, platform-independent, community-supported software for describing and comparing microbial communities. Appl Environ Microbiol.

[45] Hassanin O, Abdallah F, El-Araby IE. Molecular characterization and phylogenetic analysis of Marek’s disease virus from clinical cases of Marek’s disease in Egypt. Avian Dis. 2013;57(2 Suppl):555 – 61. 10.1637/10337-082912-Reg.1. PMID: 23901775. 10.1637/10337-082912-Reg.1 23901775

[46] Raue R, Gerlach H, Müller H (2005). Phylogenetic analysis of the hexon loop 1 region of an adenovirus from psittacine birds supports the existence of a new psittacine adenovirus (PsAdV). Arch Virol.

[47] Kumar S, Stecher G, Li M, Knyaz C, Tamura K (2018). MEGA X: Molecular Evolutionary Genetics Analysis across Computing Platforms. Mol Biol Evol.

