# Supplementary material for: Application of metagenomics for diagnosis of broilers displaying neurological symptoms
Source: BMC Vet Res. 2023 Oct 5;19:190. doi: 10.1186/s12917-023-03732-y (PMC10552438; doi:10.1186/s12917-023-03732-y)
Supplement: Supplementary file 1 — Supplementary Material 1 [file 12917_2023_3732_MOESM1_ESM.docx]

**Additional file 1**

| Pathogen | The results of PCR/RT-PCR | Reference |
| --- | --- | --- |
| *Clostridium botulinum*  (*C. botulinum*)  Avian Influenza Virus (AIV)  Avian Encephalitis Virus  (AEV) | 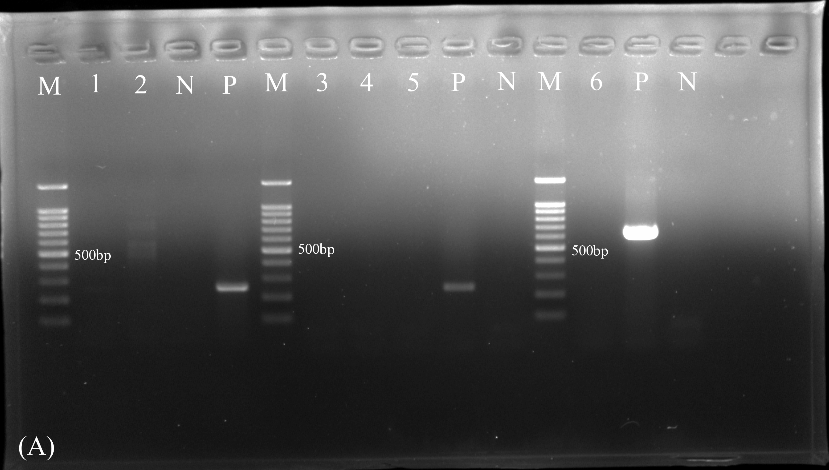 | [29-31] |
| Newcastle Disease Virus  (NDV) | 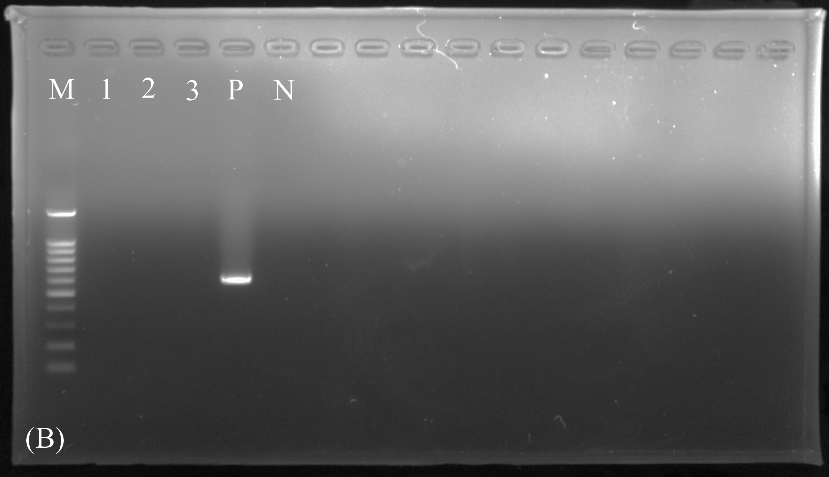 | LiliF® NDV RT-PCR Kit,  Intron biotechnology,  Korea |

Table S1. Full length of gel image for PCR and RT-PCR assays. (A) Results of PCR and RT-PCR for AIV, AEV, and *C. botulinum*. lane M: 100bp ladder, lane N: negative control, lane P: positive control, lane 1-2: the liver and intestine for *C. botulinum*, lane 3-5: the trachea, cecal tonsil, and kidney for AIV, lane 6: the brain for AEV. (B) Results of RT-PCR for NDV. lane M: 100bp ladder, lane N: negative control, lane P: positive control, lane 1-3: the trachea, cecal tonsil, and kidney for NDV.
